# A Thoroughbred

**Published:** 1905-09

**Authors:** 


### A Thoroughbred.

Our good friend, the genial and talented editor of the TEXAS MEDICAL JOURNAL, Dr. F. E. Daniel, announces the fact that his excellent journal is just entering its twenty-first year, "strong, vigorous and splendidly supported by the best men in Texas." What more can a medical editor ask for, when he has the loyal support of his friends in a chosen profession.

It puts us in mind, also, that the *New England Medical Monthly* was started just three years before this time, and is a pretty healthy young man, too, thank you, in its twenty-fourth year.

Daniel has always been outspoken and true. He has denounced fraud wherever he found it, and has always sounded the alarm with clarion notes.

The ears of the editor of the *Journal of the American Medical Association* must have burned indeed when the editorial in the June number of the 'Red Back," entitled "The Chicago Octopus," was written. It's hot stuff, and alas, alack, too true, too true, my brother. May there always be plenty of strength to Daniel's editorial elbow to continue the work twenty-one years more.—*New England Medical Monthly.*

